# HIV-2 Integrase Variation in Integrase Inhibitor-Naïve Adults in Senegal, West Africa

**DOI:** 10.1371/journal.pone.0022204

**Published:** 2011-07-12

**Authors:** Geoffrey S. Gottlieb, Robert A. Smith, Ndeye Mery Dia Badiane, Selly Ba, Stephen E. Hawes, Macoumba Toure, Alison K. Starling, Fatou Traore, Fatima Sall, Stephen L. Cherne, Joshua Stern, Kim G. Wong, Paul Lu, Moon Kim, Dana N. Raugi, Airin Lam, James I. Mullins, Nancy B. Kiviat, Papa Salif Sow for the UW-Dakar HIV-2 Study Group

**Affiliations:** 1 Department of Medicine, School of Medicine, University of Washington, Seattle, Washington, United States of America; 2 Department of Pathology, School of Medicine, University of Washington, Seattle, Washington, United States of America; 3 Clinique des Maladies Infectieuses Ibrahima DIOP Mar, Centre Hospitalier Universitaire de Fann, Universite Cheikh Anta Diop de Dakar, Dakar, Senegal; 4 Department of Epidemiology, School of Public Health and Community Medicine, University of Washington, Seattle, Washington, United States of America; 5 Department of Microbiology, School of Medicine, University of Washington, Seattle, Washington, United States of America; George Mason University, United States of America

## Abstract

**Background:**

Antiretroviral therapy for HIV-2 infection is hampered by intrinsic resistance to many of the drugs used to treat HIV-1. Limited studies suggest that the integrase inhibitors (INIs) raltegravir and elvitegravir have potent activity against HIV-2 in culture and in infected patients. There is a paucity of data on genotypic variation in HIV-2 integrase that might confer intrinsic or transmitted INI resistance.

**Methods:**

We PCR amplified and analyzed 122 HIV-2 integrase consensus sequences from 39 HIV-2–infected, INI-naive adults in Senegal, West Africa. We assessed genetic variation and canonical mutations known to confer INI-resistance in HIV-1.

**Results:**

No amino acid-altering mutations were detected at sites known to be pivotal for INI resistance in HIV-1 (integrase positions 143, 148 and 155). Polymorphisms at several other HIV-1 INI resistance-associated sites were detected at positions 72, 95, 125, 154, 165, 201, 203, and 263 of the HIV-2 integrase protein.

**Conclusion:**

Emerging genotypic and phenotypic data suggest that HIV-2 is susceptible to the new class of HIV integrase inhibitors. We hypothesize that intrinsic HIV-2 integrase variation at “secondary” HIV-1 INI-resistance sites may affect the genetic barrier to HIV-2 INI resistance. Further studies will be needed to assess INI efficacy as part of combination antiretroviral therapy in HIV-2–infected patients.

## Introduction

HIV-2 is endemic in West Africa and has limited spread to a number of other locales worldwide [1]. Compared to HIV-1, HIV-2 infection is characterized by a much longer asymptomatic stage, lower plasma viral loads, slower decline in CD4 counts, decreased mortality rate due to AIDS, and lower rates of genital tract shedding, mother to child transmission, and sexual transmission [1], [2], [3], [4], [5], [6], [7]. Nonetheless, a significant proportion of HIV-2–infected individuals eventually progress to AIDS and may benefit from antiretroviral therapy (ART) [3], [8]. Treatment of HIV-2 infection is complicated by the intrinsic resistance of the virus to non-nucleoside reverse transcriptase inhibitors (NNRTIs) and the fusion inhibitor T-20 (enfuvirtide) [9], [10]. In addition, HIV-2 exhibits a low genetic barrier to nucleoside reverse transcriptase inhibitor (NRTI) resistance and is partially resistant to several protease inhibitors (PIs) [9], [10], [11], [12], [13].

In preliminary studies, the integrase inhibitors (INIs) raltegravir, elvitegravir and S/GSK1349572 have shown *in vitro* activity against a limited number of wild-type HIV-2 strains [14], [15], [16] and genotypic surveys from Europe suggest that primary mutations leading to INI resistance in HIV-1 are rare in HIV-2 sequences from INI-naïve individuals [15], [17], [18]. Anecdotal clinical reports suggest that raltegravir-containing regimens can initially suppress HIV-2 plasma RNA loads and may therefore be useful for treating HIV-2 infection [19], [20], [21], [22], [23], [24], [25]. However, the long-term therapeutic benefits of raltegravir and other INIs will likely be compromised by emergent drug resistance, as evidenced by the appearance of resistance-associated mutations in sequences from raltegravir-treated HIV-2 patients [19], [20], [21], [22], [23], [24], [25]. Although a small sampling of patient-derived and *in vitro*-selected HIV-2 variants have been characterized, the effects of specific amino acid changes in HIV-2 integrase on INI susceptibility are largely unknown and may potentially be modulated by other naturally-occurring amino acids in the HIV-2 integrase protein.

With these issues in mind, we examined the prevalence of INI resistance-associated mutations in HIV-2 sequences from INI-naïve patients in Senegal, West Africa. To our knowledge this is the first such genotypic survey of potential INI-resistance from HIV-2 endemic West Africa. Our results suggest that intrinsic INI resistance is infrequent in West-African HIV-2 isolates, but that background variation in HIV-2 integrase may accelerate the development of INI resistance in this patient population.

## Methods

### Subjects and specimen collection

Patient samples were collected as part of an NIH-sponsored, ongoing prospective longitudinal cohort study of ART for HIV-2 infection in Senegal, West Africa; enrollment began in October 2005 [26]. HIV-2 infected individuals with clinical AIDS, CD4 counts <200/mm^3^, or <350/mm^3^ with clinical symptoms were treated with antiretrovirals as part of the Senegalese Government Antiretroviral program (ISAARV) at the Clinique des Maladies Infectieuses Ibrahima DIOP Mar, Centre Hospitalier Universitaire de Fann, Universite Cheikh Anta Diop de Dakar, Dakar, Senegal. HIV-2–infected subjects initiating ART (and those already on ART) in the ISAARV program were referred to participate in this study.

Subjects were screened for HIV-1 and HIV-2 by serologic testing. Serum samples were tested for HIV seropositivity using a microwell plate HIV-1/HIV-2 enzyme immunoassay (Genetic Systems) or Genscreen HIV-1/2 (BioRad) or by a rapid HIV test (Determine; Inverness Medical). HIV-2 seropositivity was confirmed using a rapid synthetic peptide-based membrane immunoassay (Multispot; Sanofi Pasteur) or the ImmunoComb II HIV-1 & 2 BiSpot assay (Orgenics), both of which distinguish HIV-1, HIV-2 and dual HIV-1/HIV-2 seropositivity. At enrollment and subsequent follow-up visits (at 1 month and then every 4 months), subjects underwent a physical examination and completed an interview with questions concerning demographic characteristics and sexual and other behaviors. A routine medical history and exam was performed and recorded on a standardized form by study clinicians. Peripheral blood was collected into tubes containing ethylenediaminetetraacetic acid (EDTA) and analyzed using a FACSCount analyzer (Becton Dickinson Biosciences, San Jose, CA) to determine the number of CD4, CD8 and CD3 cells/mm^3^. Samples were also subjected to HIV-2 quantitative RNA viral load assays as previously described [27].

### HIV-2 Integrase genotyping

Purified PBMC DNA was extracted from each patient sample using the QIAamp DNA Blood Mini Kit (Qiagen Inc., Valencia, CA) and quantified by spectrophometry (Nanodrop, Wilmington, DE). HIV-2–specific nested PCR [26], [27] was used to amplify the integrase-encoding region of *pol* (nucleotides 4738 to 5777, numbered as per HIV-2_ROD_, GenBank accession # M15390). Briefly, PCR was performed using 0.1–0.25 micrograms of PBMC DNA per reaction. Nested primers for PCR amplification were as follows: first round forward, H2AB_INT F1 (AAR GAA GCA RTM TAT GTW GSA TGG GTS CCA GC); first round reverse, H2AB_INT R1 (GGA CAA TAW CTT TTC YCC YCT GAT GGC TCT YCT TAC TTC); second round forward, H2AB_INT F2 (CAG GAA GTA GAY CAY TTA GTR AGT CAR GG); second round reverse, H2AB_INT R2 (GGG AAT ATT ACY CTR CTG CAA GTC CAC C). Reaction conditions and thermal cycling parameters were as previously described [26], [27].

All PCR amplifications were performed using procedural safeguards to prevent contamination including aliquoting of all reagents and physical separation of sample processing and post-PCR handling steps. In addition, negative control reactions that lacked template DNA were included in every PCR experiment, and reactions containing 10 genome equivalents of HIV-2 DNA (pROD10) were used to monitor PCR amplification efficiency. Bulk PCR products were agarose gel-purified (S.N.A.P. UV-Free Gel Purification Kit, Invitrogen) and sequenced via standard dideoxy- chain termination methods using primers H2AB_INT_seqR (AAATTCATGCAATGAACTGCC), H2AB_INT_seqF (TAGTAGAAGCAATGAATCACC), H2AB_INT F2 and H2AB_INT R2 (see above). Genbank Accession numbers of the HIV-2 sequences from this study: JF811132-JF811253.

### Phylogenetic analyses and resistance-associated genotyping

All patient-derived sequences were assessed for potential sample mix-up and contamination as recommended by the Division of AIDS, National Institute of Allergy and Infectious Diseases, National Institutes of Health (http://www.hiv.lanl.gov/content/sequence/TUTORIALS/CONTAM/contam_main.html). HIV-2 patient sequences were aligned with reference sequences from the Los Alamos National Laboratory HIV Database (http://hiv-web.lanl.gov) using Muscle (http://www.ebi.ac.uk/Tools/msa/muscle) followed by manual adjustment using MacClade (version 4.08). Neighbor-joining and maximum likelihood methods were used to estimate phylogenetic trees with PAUP* (v4.0 b10) and DIVEIN (http://indra.mullins.microbiol.washington.edu/DIVEIN). HIV-2 sequences were assigned to groups (“subtypes”) based on these phylogenetic analyses. In addition, the integrase-encoding region of each patient sequence was translated in MacClade (version 4.08), and amino acid variation was assessed at sites associated with INI resistance in HIV-1. The list of integrase sites analyzed in our study was compiled from the Stanford HIV Drug Resistance Database (http://hivdb.stanford.edu/cgi-bin/INIResiNote.cgi) and the International AIDS Society-USA Update of Drug Resistance Mutations in HIV-1 (December 2010; http://www.iasusa.org/resistance_mutations/mutations_figures.pdf).

### Ethics Statement

This study was conducted according to procedures approved by the Institutional Review Boards of the Universities of Washington and Dakar, and the Senegalese National AIDS Committee-Ministry of Health. All subjects provided written informed consent for study participation.

## Results

**Figure 1:**
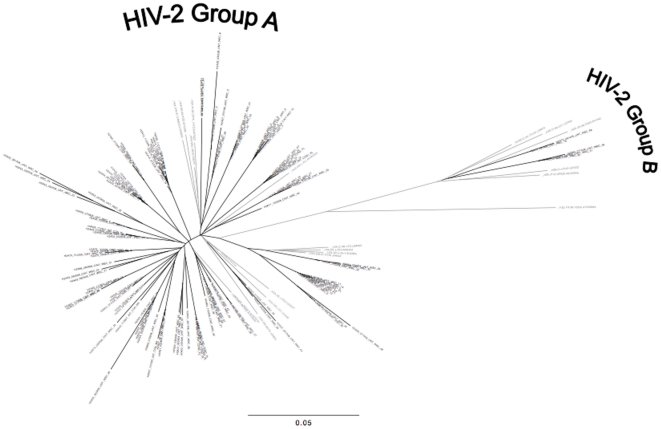
Phylogenetic tree of HIV-2 integrase nucleotide sequences (N = 122, black taxa) from 39 INI-naïve Senegalese adults and HIV-2 reference sequences (gray taxa) from the Los Alamos HIV Database ( http://www.hiv.lanl.gov **).** HIV-2 Group A (N = 37 subjects) and Group B (N = 2 subjects) clades are shown.

We analyzed HIV-2 integrase sequences from 39 Senegalese adults whose baseline characteristics are shown in Table 1. All individuals were INI-naïve; 6 were ART-naïve, and 33 patients were receiving NRTI+PI–based regimens at the time of integrase genotyping (median time on ART: 711 days, range: 90–2642 days). Median HIV-2 plasma viral loads and CD4 counts at time of sampling were 2.76 log_10_ copies/ml and 225 cells/mm^3^, respectively. A total of 122 HIV-2 patient-derived consensus (“bulk”) sequences were obtained, with longitudinal sequences from 24 (62%) individuals. Of the 39 patients in our study, 37 were infected with group A HIV-2, and two were infected with group B virus; no intra-group HIV-2 recombinants or dual infections (intra- or inter-group) were detected (Figure 1).

**Table 1:** Baseline characteristics of HIV-2 infected Patients.

N = 39	
**Females** (%)	28 (72%)
**Age** (median, range)	48 (22, 61)
**WHO Stage**	
1	6 (15%)
2	8 (21%)
3	18 (46%)
4	7 (18%)
**Baseline CD4 count** (cells/mm^3^; Median, Range)	225 (6, 819)
**Baseline HIV-2 Plasma RNA**Median (log_10_ copies/mL)(Range, % undetectable (<25 copies/mL))	2.76 (1.63, 4.12, 38%)

**Figure 2:**
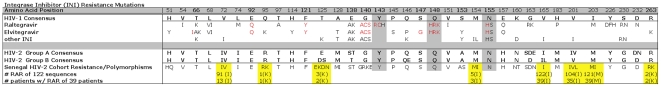
HIV-2 integrase variation at sites implicated in INI resistance. (Top) Amino acid substitutions that appear in HIV-1 sequences from patients treated with raltegravir, elvitegravir, or other INIs, or that emerge in HIV-1 in response to INI selection in culture. This list was compiled from sources listed under Methods. The consensus HIV-1 sequence (http://www.hiv.lanl.gov) is shown for comparison. Gray boxes indicate the locations of primary INI resistance mutations in HIV-1. Amino acid changes that are associated with >5-fold resistance to raltegravir or elvitegravir in HIV-1 (http://hivdb.stanford.edu) are shown in red. (Bottom) Consensus sequences for group A and group B HIV-2 isolates were derived from previously-published data (http://www.hiv.lanl.gov); positions that contain two or more residues are polymorphic in HIV-2. Patient-derived HIV-2 integrase sequences (n = 122) were obtained from 39 INI-naïve individuals in the Senegal cohort. HIV-2 integrase residues that correspond to INI resistance-associated mutations in HIV-1 are highlighted in yellow boxes. Values below the boxes indicate the number of HIV-2 patient sequences containing the each resistance-associated residue (RAR, in parentheses) and the number of patients in which these amino acids were present.

To assess the potential for intrinsic INI resistance in our HIV-2 cohort, we determined the amino acid sequence encoded by each patient-derived PCR product and examined the specific residues present at each of 35 individual INI resistance-associated sites in the integrase protein (Figure 2). Importantly, at the three positions known to be pivotal for INI resistance in HIV-1 (143, 148 and 155), all of the Senegalese HIV-2 sequences were identical to the consensus HIV-1 genotype (*i.e.*, no amino acid differences relative to the HIV-1 consensus sequence were observed at these sites). However, we observed a large number of differences between HIV-1 and HIV-2 at other sites implicated in INI resistance. Amino acids corresponding to resistance-associated changes in HIV-1 were detected at positions 72I, 95K, 125K, 154I, 165I, 201I, 203M, and 263K of the HIV-2 integrase protein (Figure 2, yellow boxes). The most common resistance-assocated residues were 72I, 165I, 201I and 203M (Figure 2, bottom). Taken together, these findings demonstrate that HIV-2 sequences from INI-naïve patients in Senegal frequently encode amino acids that correspond to “secondary” resistance-associated changes in HIV-1.

## Discussion

In our current study of HIV-2 integrase-encoding sequences from INI-naïve patients in Senegal, we found no evidence of the primary mutations responsible for raltegravir or elvitegravir resistance in HIV-1. Although we cannot rule out the possibility that Y143R/C, Q148H/R/K or N155H variants exist at low frequencies in HIV-2 patients, our data indicate that such mutants are rarely present as the majority genotype in INI-naïve individuals from the Senegal cohort. In contrast, we identified several naturally-occurring amino acids in HIV-2 integrase that are equivalent to secondary raltegravir or elvitegravir resistance-associated replacements in HIV-1. These data raise the possibility that HIV-2 variants containing primary INI resistance mutations may exhibit higher levels of INI resistance and/or improved replication fitness relative to their HIV-1 counterparts. Accordingly, the genetic barrier to raltegravir or elvitegravir resistance may be lower in HIV-2 relative to HIV-1, as shown in recent studies of NRTI-resistant viruses [13], [28]. Additional culture-based studies of patient-derived strains and site-directed mutants of HIV-2 integrase are required to address this issue.

Our analysis of West African patients, together with previous studies of HIV-2–infected individuals residing in Europe [15], [17], [18], provides encouraging information suggesting that intrinsic or transmitted INI-resistance in HIV-2 is rare at the present time. However, studies of a limited number of raltegravir-treated patients suggest that INI resistance is likely to be a growing clinical problem in HIV-2, and that the canonical INI-resistance pathways defined by replacements Y143R/C, Q148H/R/K and N155H in HIV-1 integrase may play similar roles in HIV-2 [19], [20], [21], [22], [23], [25]. In this regard, the recent development of novel strand transfer inhibitors with improved activity against INI-resistant mutants of HIV-1 [29], [30] may lead to favorable treatment options for HIV-2 patients harboring raltegravir- or elvitegravir-resistant viruses.

Because HIV-2 is intrinsically resistant to many of the antiretroviral drugs used to treat HIV-1, the emerging data on HIV-2 integrase variation and INI sensitivity potentially provide a new avenue for effective HIV-2 treatment. Ultimately, however, only appropriately designed randomized trials of specific antiretroviral regimens will provide the information patients and clinicians need to improve HIV-2 treatment [31], [32].
